# Long-term results of curative intraluminal high dose rate brachytherapy for endobronchial carcinoma

**DOI:** 10.1186/1748-717X-7-112

**Published:** 2012-07-23

**Authors:** Hidemasa Kawamura, Takeshi Ebara, Hiroyuki Katoh, Tomoaki Tamaki, Hitoshi Ishikawa, Hideyuki Sakurai, Takashi Nakano

**Affiliations:** 1 Department of Radiation Oncology, Gunma University Graduate School of Medicine, 3-39-22 Showa-machi, Maebashi, Gunma, 371-8511, Japan; 2 Department of Radiation Oncology, Saitama Medical University International Medical Center, 1397-1 yamane Hidaka-shi, Saitama, Japan; 3 Department of Radiation Oncology and Proton Medical Research Center, Tsukuba University, Tennodai 1-1-1, Tsukuba, Ibaraki, Japan

**Keywords:** Lung cancer, Radiation therapy, High dose rate, Intraluminal brachytherapy, Curative intent

## Abstract

**Background:**

The treatment strategy of central lung tumors is not established. Intraluminal brachytherapy (ILBT) is widely used for palliative treatment of endobronchial tumors, however, it is also a promising option for curative treatment with limited data. This study evaluates the results after ILBT for endobronchial carcinoma.

**Method:**

Sixteen-endobronchial carcinoma of 13 patients treated with ILBT in curative intent for 2000 to 2008 were retrospectively reviewed. ILBT using high dose rate 192 iridium thin wire system was performed with 5 Gy/fraction at mucosal surface. The patient age ranged from 57 to 82 years old with median 75 years old. The 16 lesions consisted of 13 central endobronchial cancers including 7 roentgenographically occult lung cancers and 3 of tracheal cancers. Of them, 10 lesions were treated with ILBT of median 20 Gy combined with external beam radiation therapy of median 45 Gy and 6 lesions were treated with ILBT alone of median 25 Gy.

**Results:**

Median follow-up time was 32.5 months. Two-year survival rate and local control rate were 92.3% and 86.2%, respectively. Local recurrences were observed in 2 lesions. Three patients died due to lung cancer (1 patient) and intercurrent disease (2 patients). Complications greater than grade 2 were not observed except for one grade 3 dyspnea.

**Conclusions:**

ILBT combined with or without EBRT might be a curative treatment option in inoperable endobronchial carcinoma patients with tolerable complication.

## Background

With the recent advance in diagnostic techniques, increasing number of small lung tumors has been detected. In particular, widespread use of bronchoscopy and sputum cytology detects tumors localized in the bronchial lumen. Surgery is regarded as the first choice of treatment for endobronchial carcinoma. However, endobronchial carcinoma is usually detected in high-risk patient and it is not rare that the patient has poor pulmonary function. Furthermore, 10% of patients have synchronous or non-synchronous multiple lesions [1,2]. Because of the poor pulmonary function and multiple lesions, curative surgery is not always indicated for endobronchial carcinoma. Thus, treatment strategy for early endobronchial cancer in medically inoperable patients should be examined.

Intraluminal brachytherapy (ILBT) is widely performed in medically inoperable endobronchial carcinoma as a palliative and second line treatment [3-6]. On the other hand, some reports showed high response rates and the potential of curative treatment of ILBT in selected patients [7-13]. Hence, we speculate that ILBT combined with external beam radiation therapy (EBRT) may play an important role as a curative treatment for endobronchial carcinoma. Therefore, we analyze clinical result of our institute to elucidate to curability of ILBT for early lung cancer.

## Methods

The charts of 16 lesions with 13 endobronchial carcinoma patients treated with ILBT in 2000 to 2008 were retrospectively reviewed. The extent of tumor involvement was assessed by bronchoscopy, chest X-ray, chest computed tomography (CT) using contrast medium. Distant metastasis was detected by brain CT or magnetic resonance imaging, chest and abdomen CT and bone scintigraphy. Roentgenographically occult early endbronchial cancer (ROEC) was defined by Japanese lung cancer society [14]. Namely, ROEC is defined as follows; The tumor can not be detected with X-ray and CT scans, located in sub segmental or more proximal bronchi, size is less than 2 cm, peripheral margin is visible bronchoscopically, and is identified as squamous cell carcinoma histologically.

### Radiation technique and radiation dose

Radiotherapy consisted of a combination of external beam radiotherapy and intraluminal brachytherapy in principle.

#### Intraluminal brachytherapy (ILBT)

The pretreatment of ILBT was performed according to the standard procedures for bronchoscopic examination. The applicator catheter was inserted by the oral insertion method as following. The catheter was guided to the target bronchus under bronchoscopy. The catheter has two wings at the tip of the external tube (Create Medic Co. Ltd., Japan). We used 20 mm catheters for trachea, and 15 mm catheters for bronchus. After the tumor was confirmed to lie between the two wings, the wings open and act as spacers to keep the source placing at the center of the bronchus, thus the catheter was fixed [11]. The radiation source tube was inserted to replace the inner tube, and ILBT was conducted using the high dose rate ^192^Ir thin wire remote after loading system (Microselectron, Nucletron, The Netherlands). The dose distribution was calculated with orthogonal X-ray films. The irradiation dose was measured by taking the bronchial mucosal surface as the reference point, and a dose of 5 Gy per fraction was delivered at the point, two fractions per week. The distances between the radiation source and the mucosal surface were provisionally standardized of 10 mm for the trachea, 7 mm for the main bronchi, 5 mm for the lobar and segmental bronchi and 3 mm for sub-segmental bronchi from the center of the source. We confirmed the catheter positions carefully before and after each treatment to check eccentricity of the catheter by fluoroscopy.

#### External body radiation therapy (EBRT)

EBRT was performed in 10 lesions. The radiation fields were encompassed the primary lesion and regional lymph nodes area and the lesions were irradiated by anterior and posterior parallel-opposed fields. Either 6- or 10-MV X-rays were used at a dose of 2 Gy per fraction, 5 times per week.

### CT based dosimetric evaluation

CT based dosimetry was performed in the latest 1 patient. CT was scanned using the same couch of fluoroscopy in the treatment room and the data were imported into a brachytherapy planning system (PLATO v13.3, Nucletron, The Netherlands). The dose distribution and dose volume histogram were calculated.

### Follow up and statistics

Actuarial analysis was used to determine overall survival and local control by the method of Kaplan-Meier [15] using jmp version 8.0.1 (SAS Institute Inc. USA). Overall survival was measured from the first day of radiation therapy on study until death of any cause. Local control was measured from the first day of radiation therapy until the date of local and/or regional recurrence. Local control was assessed by bronchoscopy, chest X-ray and chest CT conducted frequently more than every 6 months. Biopsies were performed when there were suspicious lesions. Treatment related toxicity was evaluated with Common Terminology Criteria for Adverse Events version 3.0 (National Cancer Institute).

## Results

### Patients’ characteristics

Patients’ characteristics were shown in Table 1. The age at the treatment was 57 to 82 years old with median 75 years. Of 16 lesions, 13 lesions were central (hilar) endobronchial cancers including 7 ROEC and 3 lesions were tracheal cancers. The sizes of all tumors measured by bronchoscopy were less than 2 cm. X-ray or CT could not measure the tumors. The histology of all tumors is Squamous cell carcinoma. Of 13 patients, 8 patients had multiple cancers and 4 of these patients had multiple lung cancers. The median follow-up for all patients is 32.5 months. Chemotherapy was administered to 2 patients prior to radiation therapy for metachronous lung cancer by the referred physicians.

**Table 1:** Patient Characteristics

Number of Patients	13
Age	57-82 (median 75) years old
Male/Female	13/0
Smoking Yes/No	13/0
Brinkman Index	200–2400 (median 1620)
Number of tumors	16*
Tracheal cancer	3
Central end-bronchial cancer	13
	(ROEC 7)

### Dose of radiation therapy

Treatment’s characteristics were shown in Table 2. Ten of 16 lesions were treated with combination of ILBT and EBRT. The median dose of ILBT was 20 Gy and the median dose of EBRT was 45 Gy. Six lesions were treated with ILBT alone and the median dose of ILBT was 25 Gy.

**Table 2:** Treatment characteristics

**ILBT alone**	**6 lesions**
	20-25 Gy(median 25 Gy)
	5 Gy/fraction
**ILBT + EBRT**	**10 lesions**
ILBT	5-20 Gy (median 20 Gy)
	5 Gy/fraction
EBRT	40-61 Gy/18-30 fractions (median 45 Gy)

ILBT intraluminal brachytherapy.

EBRT external body radiation therapy.

### Local control and overall survival

**Figure 1:**
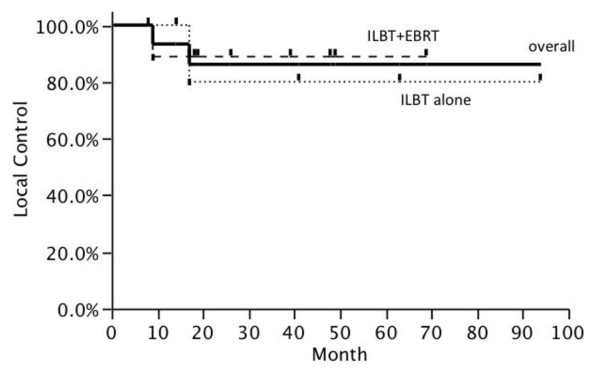
** Local control rate after the start of radiation therapy.** Kaplan-Meier estimates showing local control rates.

**Figure 2:**
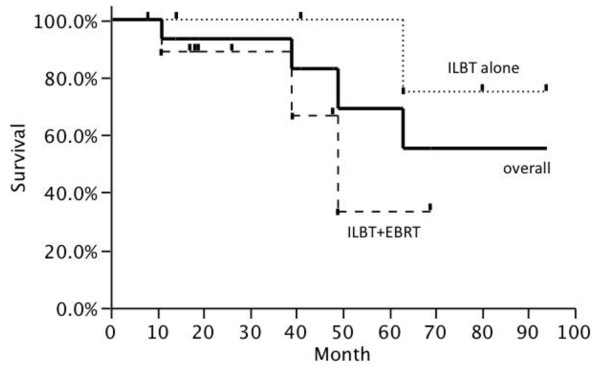
** Overall survival rate after the start of first radiation therapy.** Kaplan-Meier estimates showing overall survival rates for patients received ILBT. Three patients were treated 2 lesions each in different time point, thus, graph includes overlapping data.

The 2 year local control rate was 86.2% as shown in Figure 1. The 2 year local control rates of ROEC and others were 83.3% and 87.5%, respectively. The 2 year local control rates of ILBT alone and combination of ILBT and EBRT were 80.0% and 88.9%, respectively. The local control was not statistically different whether the lesion was ROEC or whether radiation therapy included EBRT. The 2 year overall survival rate calculated from the beginning of first radiation therapy was 92.3%. Three patients received ILBT twice for different lesions, and the total Kaplan-Meier survival curve as shown in Figure 2.

Local recurrences were observed in 2/16 lesions. One tracheal cancer patient, treated with the combination of ILBT and EBRT, died of recurrence. In another case, patient who was treated right upper lobe lesion with ILBT alone, was received salvage right upper lobe resection and is still alive without disease. The causes of death were lung cancer in 1 case and other disease in 2 cases.

### Complication

All patients had some mucosal changes (inflammations and telangiectasia), however, only two (15%) patients have clinical symptoms (developed Grade 2 or greater treatment-related complication).

One patient developed Grade 3 dyspnea. He had poor pulmonary conditions before the ILBT treatment (severe emphysema and received left upper lobe resection for atypical carcinoid and temporally received home oxygen therapy once), and promoted his past disease by treatments. Another patient developed Grade 2 cough in chronic phase. His tumor was located at the spur of left upper and lower lobe. The lesion was treated with combination of ILBT and EBRT. Four session of ILBT was performed. Of them, sources were inserted into the upper bronchus in 2 sessions and the lower bronchus in 2 sessions. EBRT was administered with 45 Gy/21 fractions after ILBT. At 19 months after treatment brochofiber scope showed the stenosis of left main bronchus.

### Case presentation of CT based dosimetric evaluation

**Figure 3:**
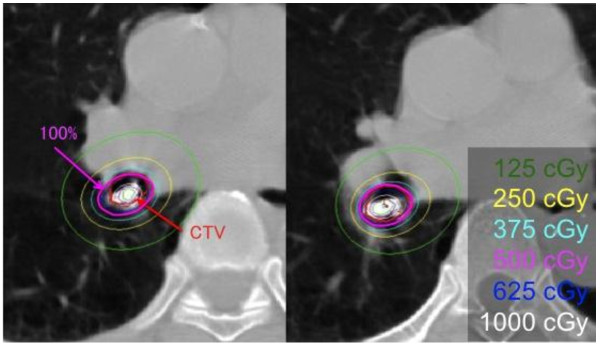
** CT based dose distribution.** A CT based dose distribution of the recent case. Red thick line is a delineated contour of clinical target volume (CTV) and magenta line indicates 100% of prescribed dose (5 Gy). 95% of the CTV receives more than 90% of the prescribed dose. The unit is cGy/fraction.

Sixty-years-old man without any symptom was diagnosed as squamous cell carcinoma of lung detected by sputum cytology. He had smoked with 1.5 packs for 45 years. Tumor located at the spur of right B6 and intermediate bronchus. ILBT was performed with 20 Gy in 4 fractions followed by EBRT of 40 Gy in 18 fractions. CT based dose distribution showed that 95% of the clinical target volume receives more than 90% of the prescribed dose (Figure 3). No remarkable acute toxicity and no recurrence were observed for a year.

## Discussion

ILBT has mainly been performed to relieve tumor-related stenosis of the trachea and/or main bronchi. Palliative-intent endobronchial ILBT has been reported to have a high response rate, and most patients who have undergone it have improved rapidly after the beginning of treatment. Many investigators therefore believe that ILBT is effective in relieving symptoms and is indicated for palliative treatment of endobronchial tumors [3-6,12,13].

On the other hand ROEC is a good candidate for curative ILBT. Some investigators used low-dose-rate intraluminal brachytherapy (LDR-ILBT) for the curative treatment of ROEC. Saito et al. evaluated a total of 79 lesions (71 cases) of ROEC, which were treated with EBRT and LDR-ILBT. The 5-year overall survival, cause specific survival, and disease-free rates were 72.3%, 96.1% and 87.3%, respectively with acceptable complications [8]. Fuwa et al. analyzed 39 ROEC patients treated with LDR-ILBT and EBRT. The therapeutic effect was complete responses in 38 patients and the 3-year and 5-year relapse-free survival rates were both 87%. They concluded that LDR-ILBT is expected to replace surgery because of less invasiveness [7]. These studies demonstrated that LDR-ILBT with EBRT could be curative therapy for ROEC.

There are few reports on high-dose-rate ILBT (HDR-ILBT) for the endobronchial cancer. Taulellue et al. adopted HDR-ILBT alone for endobronchial tumors. In sub group analysis, overall survival of Group A, which was consisted 22 (12%) patients who were medically inoperable and presented with small endobronchial tumor strictly limited to the bronchial lumen and were not treated before, was 71% at 12 months and 46% at 24 months, respectively (for all patients, 27% at 12 months and 10% at 24 months). Therefore, concluding ILBT can be tried with curative intent in patients who had small endobronchial tumors and are not candidates for other forms of therapy [13]. And Hennequin et al. showed that HDR-ILBT alone achieved a long-term cause-specific survival rate of 50% of the patients with localized endobronchial carcinoma and could be considered curative treatment [9]. According to these results, the therapeutic effects of ILBT with EBRT may be superior to that of HDR-ILBT alone even though simple comparison is difficult because of the differences of patients’ characteristics. The combination of EBRT is essential to realize tumor characteristics and homogeneities of dose distribution. There is uncertainty of the source position due to moving of catheter accompanied by breathing during the treatment. And ILBT with the rapid dose fall-off could not be enough to cover the lesion such an ROEC with a diameter more than 1 cm, which usually penetrates beyond the bronchial cartilage. Moreover, management of subclinical tumor spread including lymph node metastasis is another problem. The possibility of lymph node metastasis is un-negligible even if tumor is small. If recurrence of lymph node metastasis occur near the ILBT treatment area, re-treatment including re-irradiation or surgery is difficult. EBRT is expected a role to sterilize these lymphatic spread.

In another point of view, dosimetric and anatomical information from plain radiographs is limited. Longitudinal, tumor margins, and axial catheter displacements may occur. An incidental high dose for normal tissues could lead to severe complication while an incidental low dose for a lesion could lead to local failure. In this series, one patient developed bronchial stenosis and grade 2 cough after treatment of bronchial spur lesion. This could be a result of unexpected high dose lesion induced by the off centering of catheters due to tumor location (spur of upper and lower lobe). Furthermore, it is difficult to determine an optimal source position to the target if tumor infiltrated into bronchial spur, like the case presentation. Thus, it is important to evaluate accurately anatomical dose distribution in curative treatment. Therefore, CT based dosimetric evaluation is expected.

Studies using CT have provided valuable dosimetric information in ILBT of gynecological tumors [16], so that some institutes reports on CT based endbronchial ILBT [12,][17-19]. They pointed out that CT based dosimetry is promising, however, faster on-line treatment planning is needed for the routine clinical application of this technique. Furthermore, the rapid dose fall-off in ILBT mucosal dose prescription should be used with caution in curative treatments where ILBT, without additional external radiotherapy, is used as the sole treatment modality. The toxicity of ILBT, in particular fatal haemoptysis and bronchial wall necrosis, has been correlated with the total dose, fraction size, volume encompassed by the 100% isodose, and a proximal tumor location. Therefore CT based dosimetry is promising to evaluate these parameters. Lagerwaard et al. suggested about mucosal dose prescription, which should only be used in combination with centered applicators [18].

Because of these reasons, we have performed ILBT combined with EBRT for ROEC. Although there is no significant difference of local control and survival between ILBT alone and ILBT combined with EBRT in this study, we recommend ILBT combined with EBRT for ROEC as the reasons mentioned above. Accurate diagnosis of tumor spread with fluorescent endoscopy and endoscopic ultrasound and accurate dosimetry with CT assisted ILBT would make ILBT sophisticated and may omit of combination EBRT for particular tumors.

Recently, peripheral small lung cancers in medically inoperable patients have been well controlled with hypo-fractionated schedules by stereo-tactic radiotherapy (SRT). However, central lesions are not indicated of SRT because of the normal tissue complications [20]. ILBT is a promising alternative method for curative treatment of central small lesions. Furthermore, the candidate for good indication of ILBT may be increase. With the recent advance in diagnostic techniques, increasing number of small lung tumors has been detected. Moreover, it is not rare that patients possess several intrinsic problems including a high incidence of multiple cancers (in lungs and other organs), and a high frequency of poor pulmonary function because it commonly occurs in heavy smokers. Furthermore, elderly lung cancer patients and patients having co-morbidity are increasing and required appropriate treatment approach. Thus, medically inoperable patients are increasing and alternative approach should be investigated. The importance of ILBT as a curative intent should be re-examined because of its less invasiveness.

## Conclusions

HDR-ILBT with or without EBRT might be a curative treatment option in inoperable endobronchial carcinoma patients with tolerable toxicity.

## Abbreviations

ILBT, Intraluminal brachytherapy; EBRT, External beam radiation therapy; CT, Computed tomography; ROEC, Roentgenographically occult early endbronchial cancer; LDR, Low-dose-rate; HDR, High-dose-rate.

## Competing interests

The authors have no financial disclosures or conflicts of interest to report.

## Authors’ contribution

HK: Reviewed charts, collected data, and drafted the manuscript. TE: Reviewed charts, collected data, aided in the design of the study, reviewed the manuscript. HK: Aided in the design of the study, reviewed the manuscript. TT: Aided in the design of the study, reviewed the manuscript. HI: Aided in the design of the study, reviewed the manuscript. HS: Aided in the design of the study, reviewed the manuscript. TN: Aided in the design of the study, edited the manuscript. All authors read and approved the final manuscript.
